# Real-life evaluation of the effectiveness of biologics for chronic rhinosinusitis with nasal polyps in Europe: a Delphi study to define key variables for the INVENT registry

**DOI:** 10.3389/falgy.2025.1680703

**Published:** 2025-10-13

**Authors:** Vibeke Backer, Eugenio De Corso, Geoffrey Mortuaire, Emmanuel Prokopakis, Anette Drøhse Kjeldsen, Philippe Gevaert, Adam M. Chaker, Luisa Azevedo, Christian von Buchwald, Emilie Bequignon, Eliza Brozek-Madry, Jannis Constantinidis, Marjolein Cornet, Wytske J. Fokkens, Peter G. Gibson, Aleksander Grande Hansen, Joaquim Mullol, Sietze Reitsma, Sanna Toppila-Salmi, Michael B. Soyka, Martin Wagenmann, Peter W. Hellings

**Affiliations:** 1 Department of Otorhinolaryngology, Head & Neck Surgery, and Audiology, Rigshospitalet, Copenhagen, Denmark; 2 Unit of Otorhinolaryngology and Head-Neck Surgery, A Gemelli University Hospital Foundation IRCCS, Rome, Italy; 3 Otorhinolaryngology – Head and Neck Department – U1286 – INFINITE – Institute for Translational Research in Inflammation, Huriez Hospital, Lille, France; 4 Department of Otorhinolaryngology, University of Crete School of Medicine, Heraklion, Greece; 5 Laboratory of Translational Otorhinolaryngology Research, University of Crete School of Medicine, Heraklion, Greece; 6 Department of Otorhinolaryngology Head and Neck Surgery, Odense University Hospital, Odense, Denmark; 7 Department of Clinical Research, University of Southern Denmark, Odense, Denmark.; 8 Upper Airways Research Laboratory, Department of Head and Skin, Ghent University, Ghent, Belgium; 9 Department of Otorhinolaryngology and Center for Allergy and Environment, Technische Universität München, München, Germany; 10 Otorhinolaryngology Department, Unidade Local de Saúde da Região de Aveiro, Aveiro, Portugal; 11 Egas Moniz Health Alliance Academic Clinical Center, Aveiro, Portugal; 12 Institute of Clinical Medicine, Faculty of Health and Medical Sciences, University of Copenhagen, Copenhagen, Denmark; 13 Department of ENT, Head and Neck Surgery, Créteil Intercommunal Hospital and Henri Mondor University Hospital, Public Assistance - Paris Hospitals, Créteil, France; 14 University of Paris-Est Creteil, Paris, France; 15 Department of Otorhinolaryngology, National Institute of the Ministry of the Interior and Administration, Warsaw, Poland; 16 Department of Otorhinolaryngology Head and Neck Surgery Department, Aristotle University of Thessaloniki, AHEPA Hospital, Thessaloniki, Greece; 17 Department of Otorhinolaryngology, Alrijne Hospital, Leiderdorp, Netherlands; 18 Department of Otorhinolaryngology/Head-Neck Surgery, Amsterdam University Medical Center, University of Amsterdam, Amsterdam, Netherlands; 19 Centre of Excellence in Treatable Traits, College of Health, Medicine and Wellbeing, University of Newcastle, New Lambton Heights, NSW, Australia; 20 Department of Respiratory and Sleep Medicine, John Hunter Hospital, New Lambton Heights, NSW, Australia; 21 Asthma and Breathing Research Program, The Hunter Medical Research Institute, New Lambton Heights, NSW, Australia; 22 Department of Ear, Nose and Throat, Head and Neck Surgery, Lovisenberg Diaconal Hospital, Oslo, Norway; 23 Rhinology Unit and Smell Clinic, Department of Otorhinolaryngology, Hospital Clinic Barcelona, FRCB-IDIBAPS, Universitat de Barcelona, CIBERES, Barcelona, Spain; 24 Department of Otorhinolaryngology, University of Eastern Finland, Joensuu and Kuopia, Finland; 25 Department of Otorhinolaryngology, Wellbeing Services County of Pohjois-Savo, Kuopio, Finland; 26 Department of Allergology, Inflammation Center, Department of Allergology, Helsinki University Hospital and University of Helsinki, Helsinki, Finland; 27 Department of Otorhinolaryngology, Head and Neck Surgery, University Hospital Zurich, University of Zurich, Zurich, Switzerland; 28 Faculty of Medicine, University of Zurich, Zurich, Switzerland; 29 Department of Otorhinolaryngology, Düsseldorf University Hospital (UKD), Düsseldorf, Germany; 30 Department of Otorhinolaryngology, Head and Neck Surgery, UZ Leuven Campus Gasthuisberg, Upper Airways Research, University of Ghent, Leuven, Belgium; 31 Allergy and Clinical Immunology Research Group, Department of Microbiology, Immunology & Transplantation, KU Leuven, Leuven, Belgium

**Keywords:** chronic rhinosinusitis with nasal polyp, CRSwNP, type-2 inflammation, comorbididites, monoclonal antibodies, systematic assessment

## Abstract

**Background:**

Chronic rhinosinusitis with nasal polyps (CRSwNP) is a persistent inflammatory condition often associated with type 2 inflammation. While biologics are a promising treatment for patients with uncontrolled CRSwNP, real-world evidence is needed to optimize their use. The InternatioNal seVerE CRSwNP (INVENT) registry aims to consolidate data on biologic use in CRSwNP from local and national registries. This study describes the identification of mandatory and optional variables for inclusion in the INVENT registry using a modified Delphi process.

**Methods:**

A narrative literature review was performed to identify variables reported in real-world studies of biologic treatment for CRSwNP. A modified Delphi study was conducted between December 2024 and March 2025 involving 23 experts from Europe and Australia. Experts rated the clinical relevance of candidate variables in two online survey rounds using 9-point Likert scales. A positive response was defined as ≥70% of respondents rating a variable 7–9 and ≤15% rating it 1–3. Final agreement on mandatory and optional variables was reached through panel discussion. A validation survey was then conducted across registry centers to assess the feasibility of collecting the selected variables.

**Results:**

The Delphi process resulted in consensus on a core set of mandatory and optional variables across nine domains: demographics, medical history, previous and current biologic therapy, biomarkers, comorbidities, asthma, CRSwNP-specific outcomes, and follow-up variables. The validation survey confirmed that most mandatory variables were available or obtainable across participating centers, supporting the feasibility of data collection.

**Conclusions:**

This international Delphi study identified a consensus-based set of clinically-relevant and feasible variables for inclusion in the INVENT registry. The selected variables reflect current best practices in the management of CRSwNP and will enable robust comparisons of biologic effectiveness in real-world settings. The INVENT registry is well-positioned to inform treatment decisions, optimize use of biologics, and support a personalized approach to CRSwNP care.

## 1 Introduction

Chronic rhinosinusitis with nasal polyps (CRSwNP)—also known in patient facing communications as Nasal Polyp Syndrome—is a persistent inflammatory condition affecting up to 3% of the Western world (1–4). It is defined by the presence of bilateral, endoscopically visible nasal polyps combined with symptoms of nasal obstruction and/or discolored discharge with or without facial pain/pressure and a reduction or loss of smell for at least 12 weeks (5–7). CRSwNP is frequently associated with type 2 inflammation (1, 8), particularly in patients with more severe disease and significant smell impairment (9). It is often concomitant with asthma and non-steroidal anti-inflammatory drug-exacerbated respiratory disease (N-ERD), and other diseases related to eosinophilic inflammation (10–12). The burden of disease extends beyond nasal symptoms, with sleep disturbances, fatigue, and decreased quality of life (QoL) frequently reported (13–16).

Traditional treatment options for CRSwNP include saline rinses, intranasal corticosteroids (INCS), systemic corticosteroids (SCS), and endoscopic sinus surgery (ESS) (1, 5, 7). However, many patients continue to have uncontrolled upper airway disease and to experience CRSwNP disease recurrence despite these interventions (1, 5, 17). Monoclonal antibodies (biologics) such as omalizumab, mepolizumab, dupilumab, and, more recently, tezepelumab and depemokimab have demonstrated efficacy in treating CRSwNP (18–22) and are now approved or are in the process of being approved as add-on treatments for patients with severe uncontrolled disease and evidence of type 2 inflammation (1, 7, 23). Although biologics represent a paradigm shift in the treatment of CRSwNP, their high cost places a significant burden on healthcare systems, highlighting the need to optimize their use in clinical practice. Given their high cost, many governments set specific reimbursement criteria for biologics that vary between countries. As a result, significant differences in patterns of biologics prescribing may be observed across different countries. Real-world evidence is essential for identifying patients most likely to respond to treatment, for comparing the effectiveness of available biologic therapies, for defining appropriate outcome measures, and for determining the optimal timeframe for achieving remission.

Separate regional and national registries have been established across Europe, the USA, and Australia to evaluate the real-world effectiveness of biologics for treating CRSwNP. The international, not-for-profit European Forum for Research and Education in Allergy and Airway Diseases [EUFOREA (24)] is now coordinating efforts to consolidate data from these registries through the InternatioNal seVerE CRSwNP (INVENT) registry study (NCT06617754). This study aims to collect and analyze data from approximately 3,000 patients presenting with symptoms of CRSwNP, who meet the criteria for biologic therapy and have at least 6 months of follow-up data available. The overall objective is to compare results for different biologics in different registries at screening and follow-up, with the aim of improving individual patient care, enhancing the efficiency of biologic use, and advancing a personalized medicine approach for CRSwNP.

The first step in the INVENT registry was to perform a Delphi study to identify and select the most appropriate variables to include in the registry. This project convened experts in the field of CRSwNP from across Europe and Australia and aimed to build consensus on the mandatory and optional variables to be collected for patients included in the INVENT registry.

## 2 Methods

### 2.1 Narrative literature search

PubMed was searched on 10 November 2024 using the following search terms: real-world evidence OR RWE, nasal polyps, chronic rhinosinusitis with nasal polyps, CRSwNP, biologics [(((nasal polyps) AND ((CRSwNP) OR (chronic rhinosinusitis nasal polyps))) AND ((((RWE) OR (RWD)) OR (Real-world evidence)) OR (real-world data))) AND (biologics)]. Studies were deemed eligible if they were published between 2019 and 2024 and assessed real-world outcomes for patients with CRSwNP treated with any biologic and contained details of patient characteristics (baseline and/or post treatment). Articles not in English, author replies, and studies that did not report patient variables were excluded.

### 2.2 Delphi study design

A modified Delphi study was conducted between December 2024 and March 2025 using methodology adapted from the EPOS 2020 Delphi approach (5). The study comprised an initial expert panel meeting followed by a two-round Delphi conducted via online surveys. The objectives of round 1 were to identify essential variables for inclusion in the registry as well as variables that should be excluded. The objectives of round 2 were to determine whether variables that failed to reach a consensus in round 1 should be included as optional variables, to confirm essential variables and to determine the appropriate timepoints for data collection (screening, follow-up or both). The final aim of the study was to determine which of the variables identified through the Delphi process should be designated as mandatory and which as desirable for inclusion in future registries.

#### 2.2.1 Participants

The study included a design panel of six ear, nose and throat (ENT) specialists and one asthma specialist from various European countries (Belgium, Denmark, France, Germany, Greece, and Italy) and an expert voting group comprising 13 CRSwNP experts from Europe (Norway, Denmark, Poland, France, Greece, Spain, Portugal, Netherlands, Germany, Switzerland, Belgium and Finland) and a severe asthma expert from Australia. The design panel reviewed the findings from the literature review and developed the draft statements to be included in the first and second survey. The surveys were completed by the expert voting group but not the design panel. All experts were invited to participate based on their practical expertise, publishing records, national and regional recognition, and interest in the subject.

#### 2.2.2 Definition of consensus

In round 1, the voting group reviewed a list of variables and rated how essential each one was for inclusion in the INVENT registry. Responses for each variable were recorded using a 9-point Likert scale (1 = least essential, 9 = most essential). The voting group could also provide free text answers to explain their ratings and to suggest statement modifications. A “positive” response was defined by ≥70% of respondents rating the variable 7–9 and ≤15% rating it 1–3. A “negative” response was defined by ≥70% rating the variable 1–3 and ≤15% rating it 7–9. The design panel considered both the voting results and the free text comments to guide the variables taken through for consideration in round 2. In round 2, the voting experts were asked to rate variables using 9-point Likert scales for essential or mandatory variables (1 = not essential for inclusion, 9 = absolutely essential for inclusion) and optional or desirable variables (1 = not desirable for inclusion, 9 = absolutely desirable for inclusion). Some variables that received a positive result in round 1 were not revoted on in round 2.

After the second round of voting, the expert panel convened in a virtual meeting and via email discussions to reach final agreement on those variables that should be considered mandatory for inclusion in the registry, and which should be optional based on the responses obtained across rounds 1 and 2.

#### 2.2.3 Validation survey

Following the virtual meeting of the expert panel, a validation survey was distributed to all centers participating in the INVENT registry asking them to confirm whether they have or can obtain the variables that were identified by the Delphi process. The survey was sent to 25 individuals representing clinical centers participating in the registry from different countries; responses were received from 18 individuals from different countries.

## 3 Results

### 3.1 Literature review

**Figure 1:**
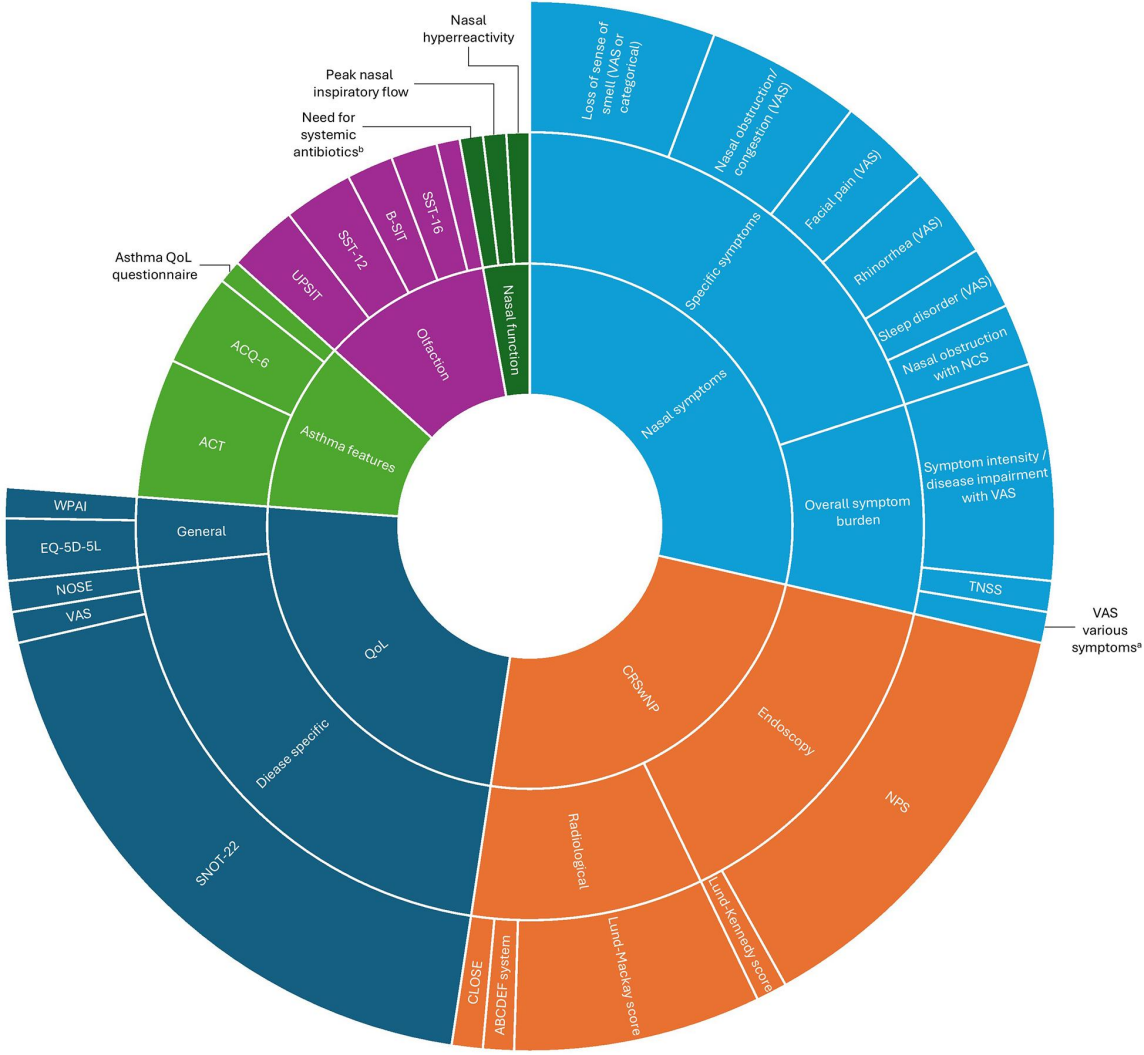
Variables/outcomes reported in published real-world studies on CRSwNP (2019–2024). ^a^Some included with individual VAS scores. ^b^Acute exacerbations of sinonasal symptoms resulting in prescription of systematic antibiotics. ACT, Asthma Control Test; ABCDEF, Anterior ethmoid artery, Basal lamella, Cribriform plate (Keros classification), Dehiscence of the lamina papyracea, Ethmoid roof asymmetry, Frontal sinus drainage pathway; ACQ-6, Asthma Control Questionnaire-6; B-SIT, Brief Smell Identification Test; CLOSE, Cribriform plate (Keros classification), Lamina papyracea, Onodi cell, Sphenoid sinus pneumatization, Ethmoid artery (anterior); EQ-5D-5l, EuroQol 5 Dimensions 5 Levels; NCS, Nasal Congestion Score; NOSE, nasal obstruction and septoplasty effectiveness; NPS, Nasal Polyp Score; QoL, quality of life; SNOT-22, sino-nasal outcome test-22; SST, Sniffin’ sticks; TNSS, total nasal symptom score; UPSIT, University of Pennsylvania Smell Identification Test; VAS, visual analog scale; WPAI, work productivity and activity impairment.

Overall, 42 publications were identified from the literature review; of which, 29 met the eligibility criteria. Multiple variables related to patient demographics, treatment center, disease history, comorbidities, biomarkers, CRSwNP disease-specific and follow-up outcomes were identified across these studies (data not shown). The CRSwNP disease-specific group included 30 different variables reported in 26 publications (Figure 1). This literature review, along with information from guidelines and other registries were used to inform the statements in the round 1 Delphi survey.

### 3.2 Delphi study

The combined results from the two voting rounds are shown in Figures 2–10 and the final mandatory and optional variables for inclusion in the INVENT registry are listed in Table 1.

**Figure 2:**
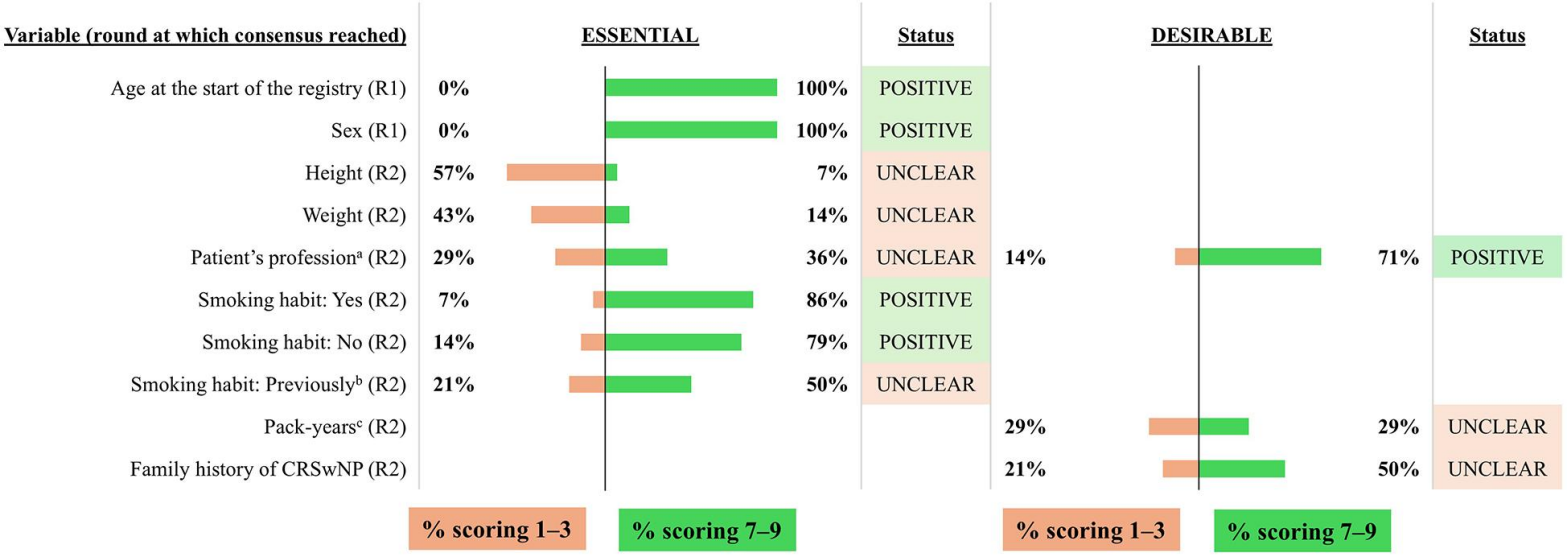
Consensus on variables/outcomes for inclusion in the INVENT registry: Demographics. R indicates the round of voting at which consensus was achieved. ^a^Profession that involves, or involved, exposure to respiratory irritants. ^b^An individual who has not smoked for the last 6 months. ^c^Average number of cigarettes per day x years of smoking/20. CRSwNP, chronic rhinosinusitis with nasal polyps.

**Figure 3:**
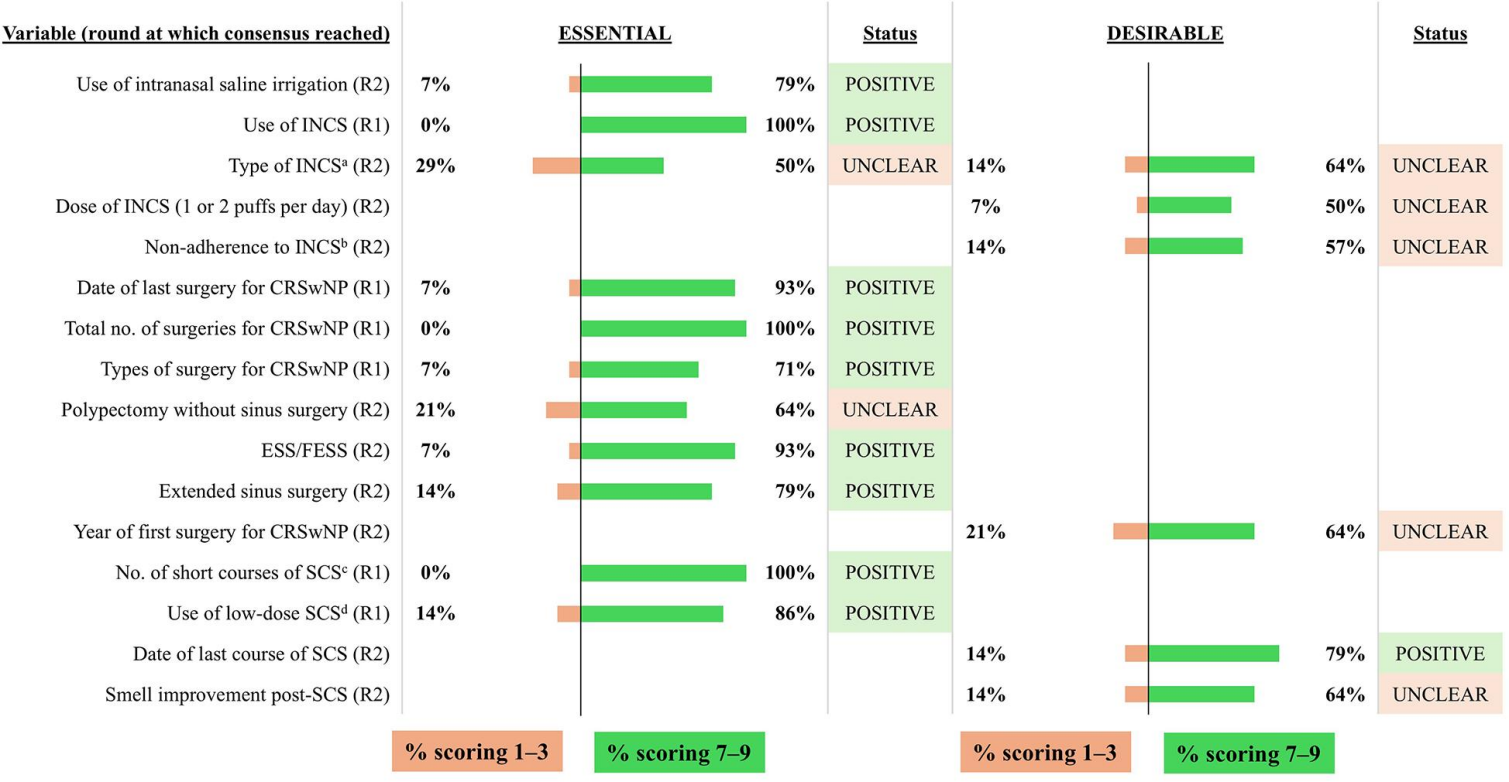
Consensus on variables/outcomes for inclusion in the INVENT registry: History of topical treatments, sinus surgery and systemic corticosteroid use. R indicates the round of voting at which consensus was achieved. ^a^Listed by international non-proprietary name. ^b^Defined as a patient who takes INCS <5 days/week (70%-80% adherence). ^c^Number of short courses (5–21 days) of SCS used for CRSwNP in the last 12 months. ^d^Use of low-dose (5–10 mg) SCS for CRSwNP in the last 12 months. CRSwNP, chronic rhinosinusitis with nasal polyps; F(ESS), (functional) endoscopic sinus surgery; INCS, intranasal corticosteroids; SCS, systemic corticosteroids.

**Figure 4:**
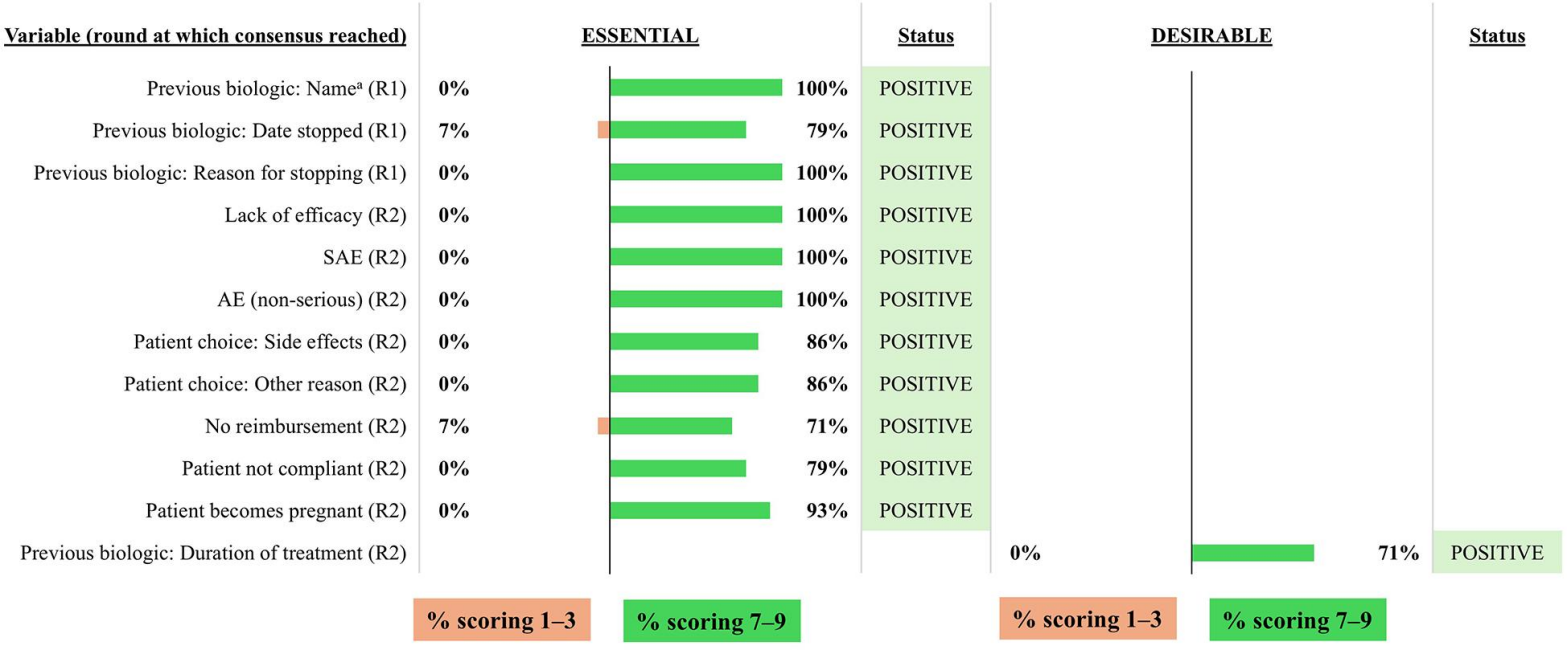
Consensus on variables/outcomes for inclusion in the INVENT registry: Previous biologic treatments. R indicates the round of voting at which consensus was achieved. ^a^Listed by international non-proprietary name. AE, adverse event; CRSwNP, chronic rhinosinusitis with nasal polyps; SAE, serious adverse event.

**Figure 5:**
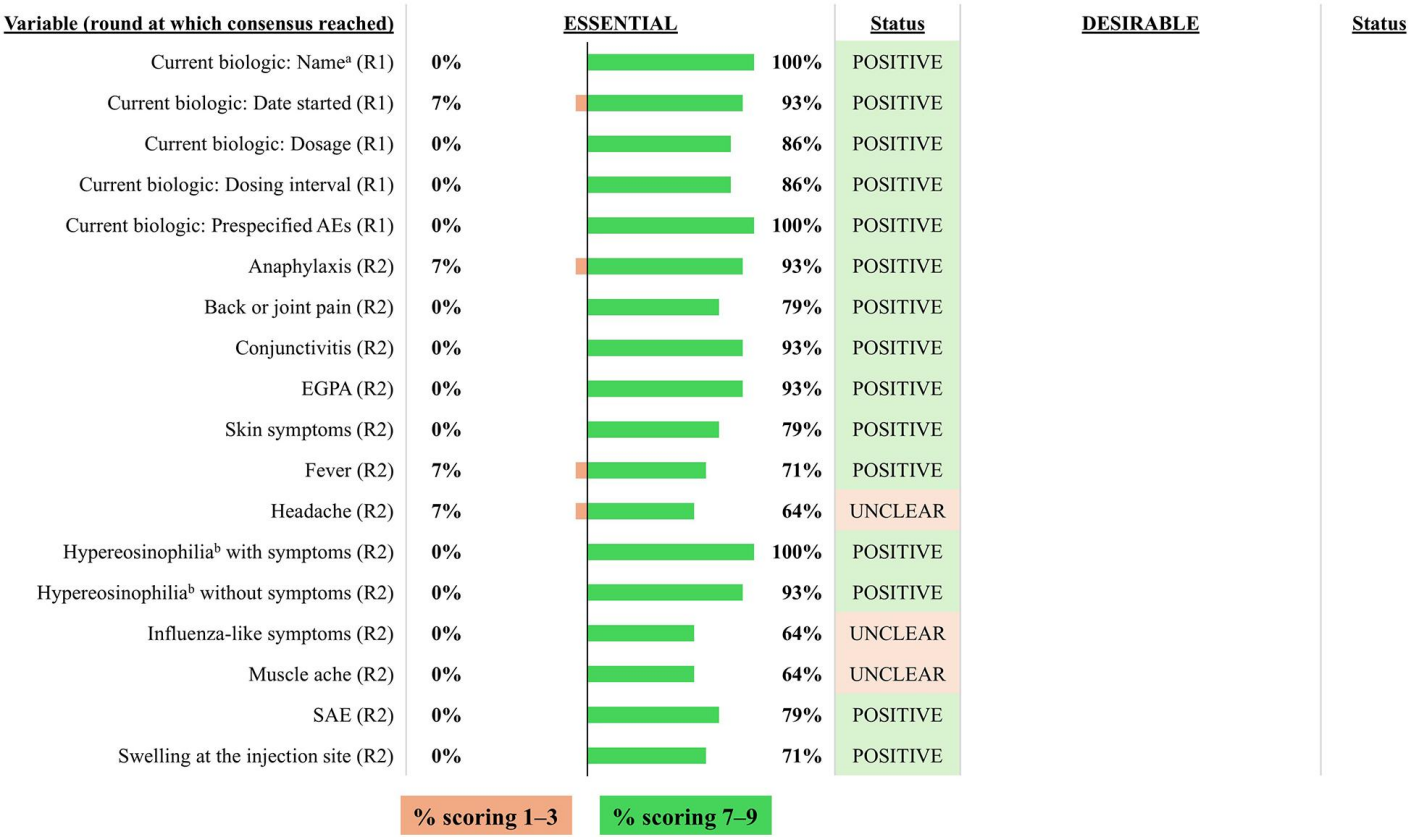
Consensus on variables/outcomes for inclusion in the INVENT registry: Current biologic treatments. R indicates the round of voting at which consensus was achieved. ^a^Listed by international non-proprietary name. ^b^Defined as >1,500 cell/*μ*l. AE, adverse event; CRSwNP, chronic rhinosinusitis with nasal polyps; EGPA, eosinophilic granulomatosis with polyangiitis; SAE, serious adverse event.

**Figure 6:**
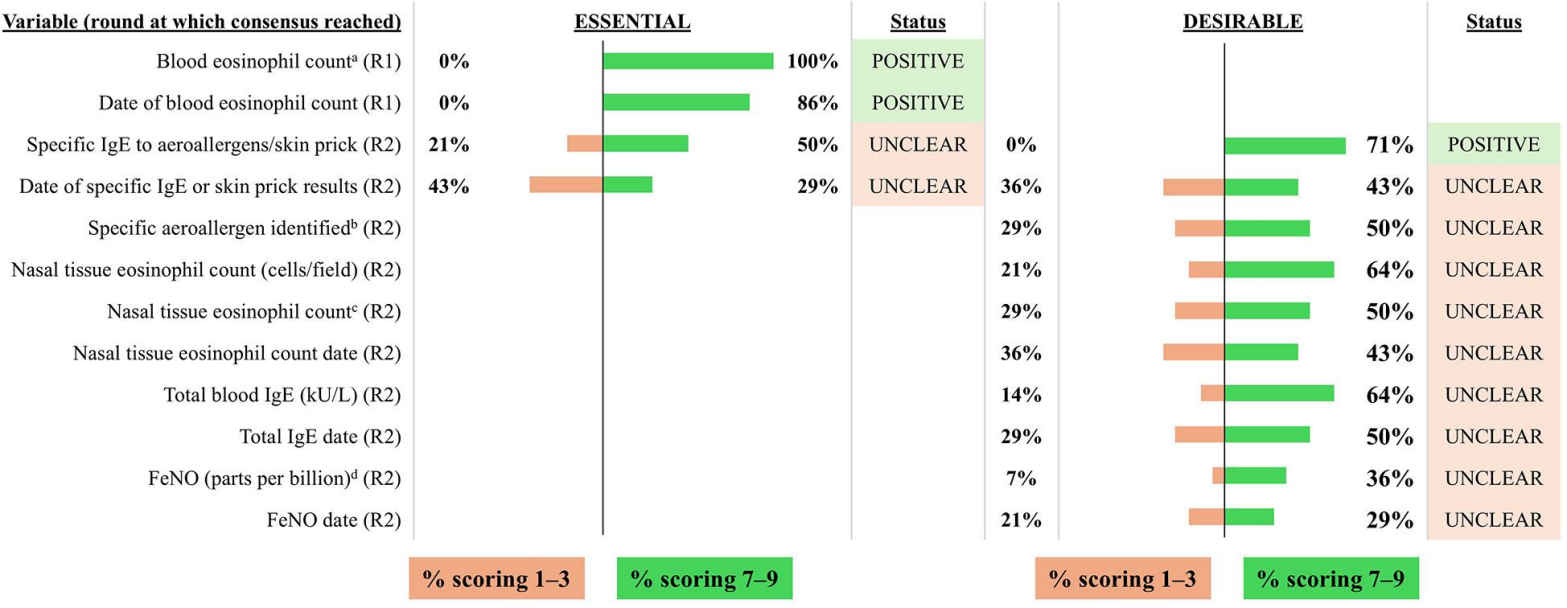
Consensus on variables/outcomes for inclusion in the INVENT registry: Biomarkers. R indicates the round of voting at which consensus was achieved. ^a^Assessed without concomitant treatment with SCS or biologics (measured as either cells/μl or as a percentage of white blood cells). ^b^Specific aeroallergen (pollen, house dust mite, pets/animals, mould, insects) to which a patient is sensitive. ^c^Assessed as either mild, moderate or severe. ^d^For patients with asthma. FeNO, fractional exhaled nitric oxide; IgE, immunoglobulin E.

**Figure 7:**
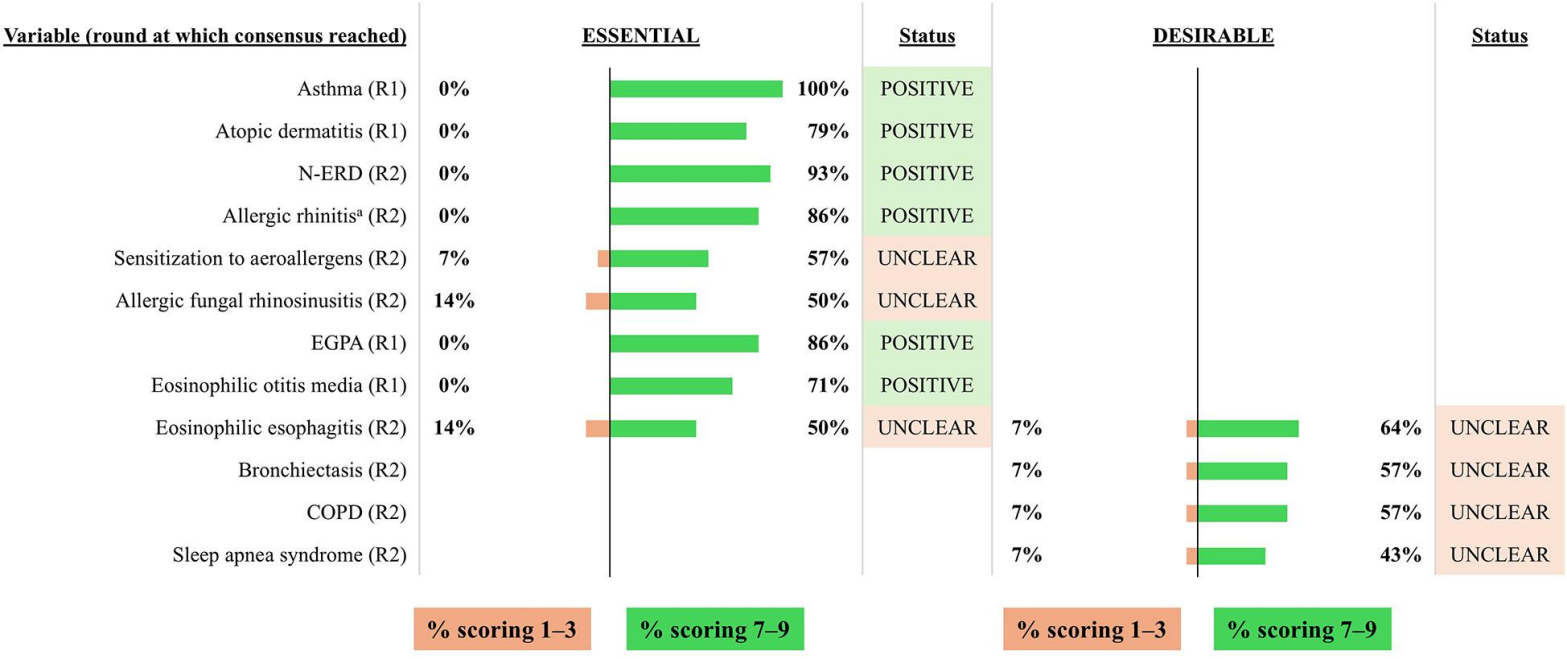
Consensus on variables/outcomes for inclusion in the INVENT registry: General comorbidities. R indicates the round of voting at which consensus was achieved. ^a^Indicating a clinically-relevant allergy to aeroallergens. COPD, chronic obstructive pulmonary disease; EGPA, eosinophilic granulomatosis with polyangiitis; N-ERD, non-steroidal anti-inflammatory drug-exacerbated respiratory disease.

**Figure 8:**
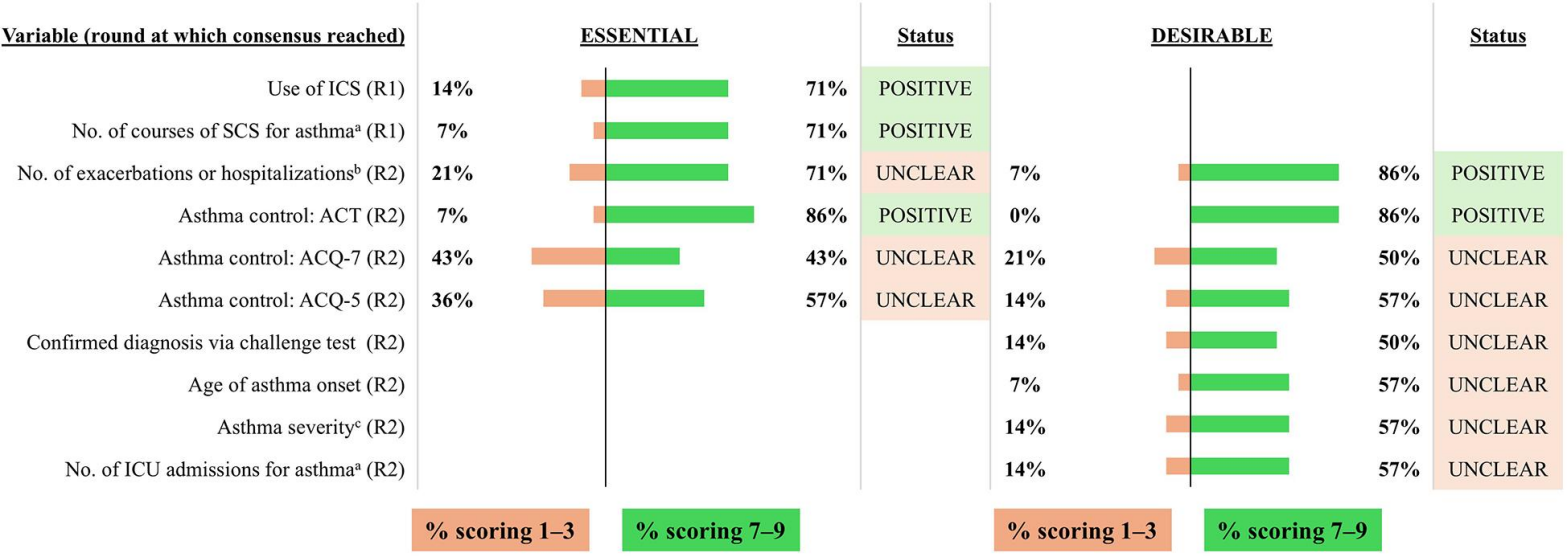
Consensus on variables/outcomes for inclusion in the INVENT registry: Asthma-related variables/outcomes. R indicates the round of voting at which consensus was achieved. ^a^Number of courses of SCS in the past 12 months. ^b^Number of exacerbations or hospitalizations due to asthma, CRSwNP or both that the patient has experienced in the past 12 months. ^c^Graded using the Global Initiative for Asthma (GINA) classification. ACT, Asthma Control Test; ACQ-5, Asthma Control Questionnaire-5; ACQ-7, Asthma Control Questionnaire-7; ICS, inhaled corticosteroids; ICU, intensive care unit; SCS, systemic corticosteroids.

**Figure 9:**
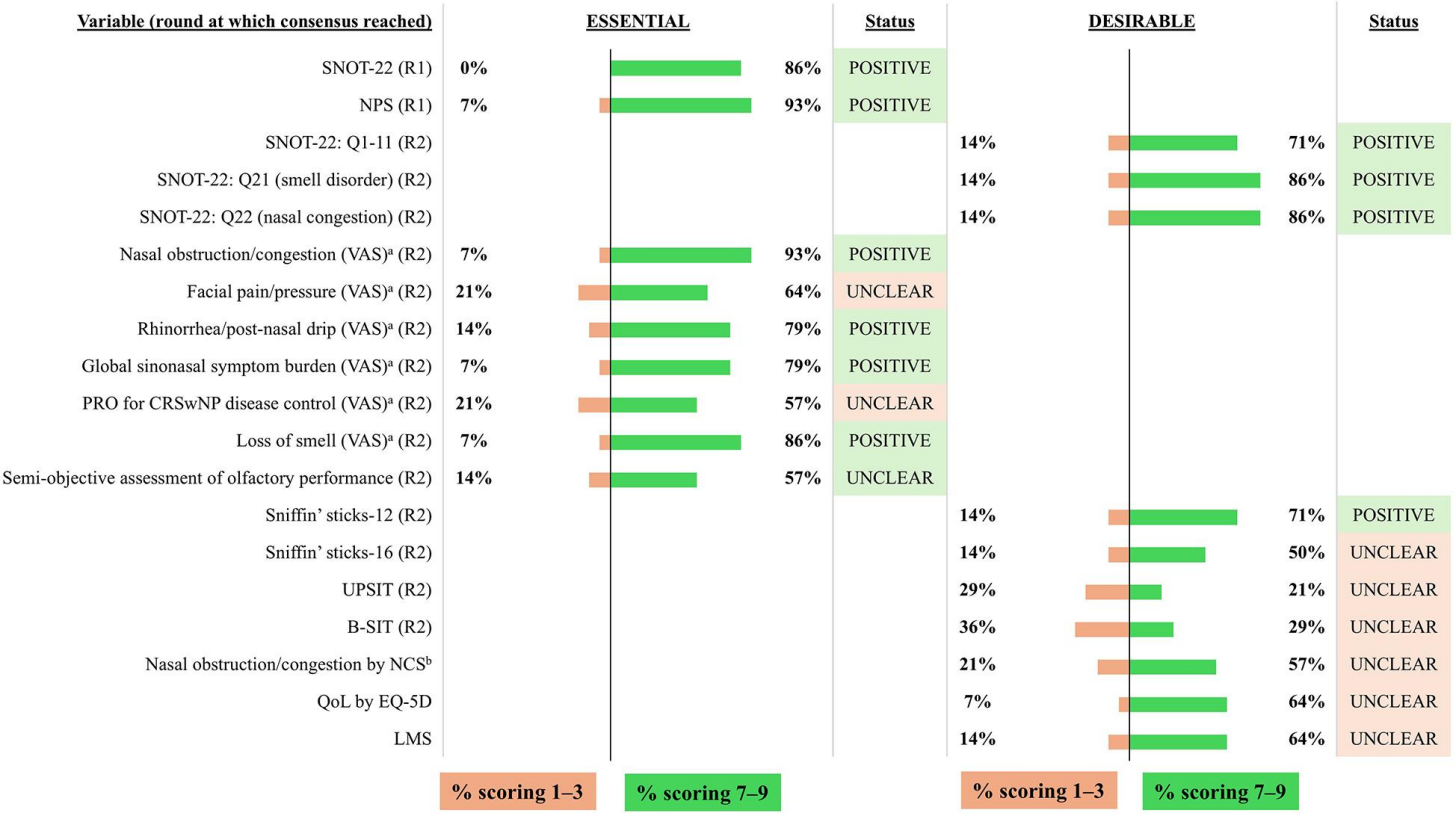
Consensus on variables/outcomes for inclusion in the INVENT registry: CRSwNP-specific variables/outcomes. R indicates the round of voting at which consensus was achieved. ^a^VAS 0–10 (where 0 is best and 10 is worst). ^b^From 0 to 3. B-SIT, Brief Smell Identification Test; CRSwNP, chronic rhinosinusitis with nasal polyps; EQ-5D, EuroQol 5 Dimensions; LMS, Lund-Mackay score; NCS, Nasal Congestion Score; NPS, Nasal Polyp Score; PRO, patient-reported outcome; Q, question; QoL, quality of life; SNOT-22, sino-nasal outcome test-22; UPSIT, University of Pennsylvania Smell Identification Test; VAS, visual analog score.

**Figure 10:**
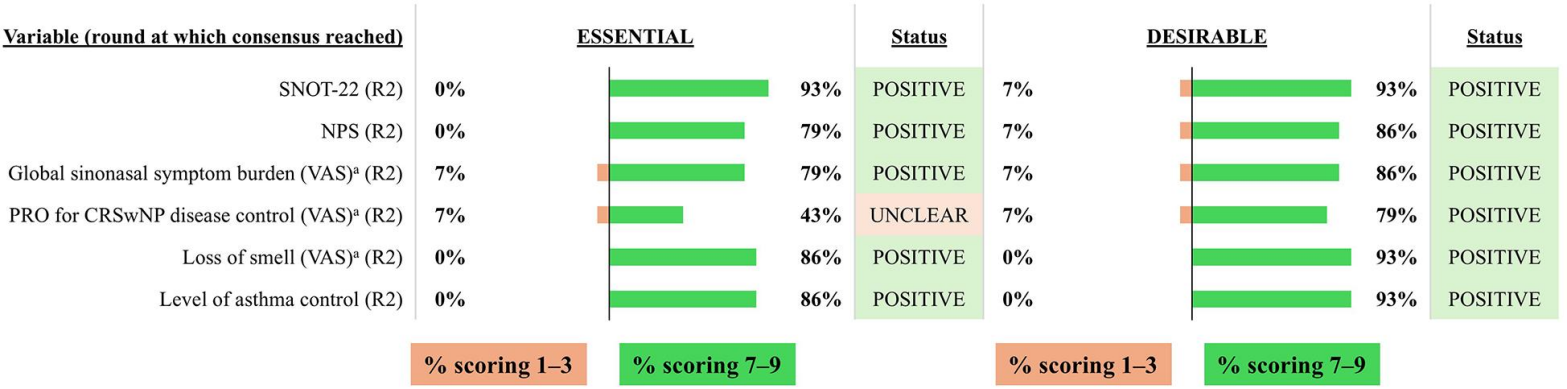
Consensus on variables/outcomes for inclusion in the INVENT registry: Follow-up variables/outcomes. R indicates the round of voting at which consensus was achieved. ^a^VAS 0-10 (where 0 is best and 10 is worst). CRSwNP, chronic rhinosinusitis with nasal polyps; NPS, Nasal Polyp Score; PRO, patient-reported outcome; SNOT-22, sino-nasal outcome test-22; VAS, visual analog score.

**Table 1:** Summary of final variables for inclusion in the INVENT registry.

Category	Mandatory variables^a^	Optional variables^b^
DEMOGRAPHICS	Age at the start of the registry^c^ Sex Smoking habit^d^	Patient's profession which involves, or involved, exposure to respiratory irritants
MEDICAL HISTORY: TOPICAL TX	Use of intranasal saline irrigation treatment for CRSwNP Use of INCS	
MEDICAL HISTORY: SURGICAL TX	Date of the patient's last surgical procedure for CRSwNP Total number of surgical procedures performed to treat a patient's CRSwNP Types of surgical procedures performed to treat a patient's CRSwNP: ESS/FESS or extended sinus surgery	
MEDICAL HISTORY: SCS	Number of short courses (5–21 days long) of SCS therapy used for CRSwNP by a patient in the last 12 months Use of low-dose (5–10 mg) SCS for CRSwNP by a patient in the last 12 months	Date (year) of the last course of SCS therapy used by the patient to treat CRSwNP
MEDICAL HISTORY: PREVIOUS BIOLOGIC TX FOR CRSwNP	International Non-proprietary Name/s of the **previous** biologic therapy/therapies used to treat the patient's CRSwNP Date when the **previous** biologic therapy used to treat the patient's CRSwNP was stopped Reason for discontinuation of **previous** biologic therapy for CRSwNP: ○ Patient becomes pregnant ○ Lack of efficacy ○ SAE ○ AE (non-serious) ○ Patient's choice due to side effects ○ Patient's choice for other reasons ○ No reimbursement ○ Patient not compliant	Duration of treatment with **previous** biologic therapies used to manage the patient's CRSwNP
MEDICAL HISTORY: CURRENT BIOLOGIC TX FOR CRSwNP	International Non-proprietary Name/s of the **current** biologic therapy/therapies used to treat the patient's CRSwNP Date when the **current** biologic therapy used to treat the patient's CRSwNP was started Dosage of the **current** biologic therapy Interval between doses of the **current** biologic therapy AEs associated with **current** biologic therapy for CRSwNP (pre-specified list): ○ Anaphylaxis ○ Back or joint pain ○ Conjunctivitis ○ EGPA ○ Fever ○ Hypereosinophilia >1,500 cells/μl with or without symptoms ○ SAE ○ Skin symptoms ○ Swelling at the injection site	AEs associated with **current** biologic therapy for CRSwNP (pre-specified list): ○ Headache ○ Influenza-like symptoms ○ Muscle ache
BIOMARKERS	Blood eosinophil count assessed without concomitant treatment with SCS or biologics (cells/μl or % of white blood cells) and date when measured	Specific IgE to aeroallergens or skin prick test results FeNO Nasal tissue eosinophil counts
GENERAL COMORBIDITIES	Asthma Atopic dermatitis N-ERD Allergic rhinitis^e^ Eosinophilic otitis media	
ASTHMA	Use of ICS as a treatment for asthma Number of courses of SCS treatments the patient has received for asthma in the previous 12 months	Number of exacerbations or hospitalizations due to asthma, CRSwNP or both that the patient has experienced in the previous 12 months Level of asthma control via ACT and/or ACQ-5/-7
CRSwNP-SPECIFIC VARIABLE	Impact of nasal symptoms measured by SNOT-22 (0–110) Polyp size and extent measured by NPS (0–8) Nasal obstruction/congestion measured by VAS (0–10 cm)^f^ Global sinonasal symptom burden measured by VAS (0–10 cm)^f^ Olfaction (loss of smell) measured by VAS (0–10 cm)^f^	Subscores of the SNOT-22 questionnaire: Q1–11 and/or Smell disorder (Q21) and/or Nasal congestion (Q22) Rhinorrhea/post-nasal drip measured by VAS (0–10 cm)^f^ Semi-objective assessment of olfactory performance using SST-12 (0–12) and/or SST-16 (0–16) and/or UPSIT (0–40) and/or B-SIT (0–12)
FOLLOW-UP	SNOT-22 (0–110) NPS (0–8) Global sinonasal symptom burden measured by VAS (0–10 cm)^f^ Olfaction (loss of smell) measured by VAS (0–10 cm)^f^ Level of asthma control (assessed with ACT or ACQ-5)	

ACQ-5, Asthma Control Questionnaire; ACT, Asthma Control Test; AE, adverse event; B-SIT, Brief Smell Identification Test; CRSwNP, chronic rhinosinusitis with nasal polyps; EGPA, eosinophilic granulomatosis with polyangiitis; FeNO, fractional exhaled nitric oxide; (F)ESS, (functional) endoscopic sinus surgery; ICS, inhaled corticosteroids; INCS, intranasal corticosteroids; NPS, Nasal Polyp Score; N-ERD, non-steroidal anti-inflammatory drug-exacerbated respiratory disease; Q, question; SAE, serious adverse event; SCS, systemic corticosteroid; SNOT-22, Sino-Nasal Outcome Test 22; SST, Sniffin’ sticks; Tx, treatment; UPSIT, University of Pennsylvania Smell Identification Test; VAS, visual analog scale.

a Core set of variables required for the registry.

b Supplementary set of variables to be entered in the registry if available.

c Calculated from date of birth and date of entry into the registry.

d Individuals who are considered not to smoke include those who have never smoked and those who have not smoked for the last 6 months.

e Indicating a clinically-relevant allergy to aeroallergens).

f Where 0 = best and 10 = worst.

#### 3.2.1 Demographics

The voting group agreed that the patient's age at the start of the registry, sex, and smoking habits were essential variables for inclusion in the INVENT registry (Figure 2). They also considered it desirable to include information on current or previous occupational exposure to respiratory irritants and the expert panel agreed to include this as an optional variable. No consensus was reached on height and weight, smoking pack-year history and family history of CRSwNP in round 2, and the expert panel agreed to exclude these from the registry. Although no consensus was reached on former smoking status, the definition of an individual who did not smoke was clarified in round 2 to include someone who has never smoked or someone who has not smoked in the last 6 months.

#### 3.2.2 Medical history

The voting group agreed that it is essential to include the use of intranasal saline irrigation and INCS in the INVENT registry (Figure 3). However, details such as the type, dose and adherence to INCS were considered neither essential nor desirable for inclusion and were therefore excluded. Regarding prior sinonasal surgeries, the date of the most recent procedure, total number of sinus surgeries, and types of surgery were deemed essential variables. The number of short courses of SCS as well as the use of low-dose SCS were also classified as essential. The date of the most recent SCS course was considered a desirable variable by the voting group and the expert panel agreed to include it as an optional variable in INVENT.

#### 3.2.3 Previous biologic therapy

For previous biologic treatments, it was considered essential to include the name of the previous biologic and date of discontinuation (Figure 4). The duration of the treatment with the previous biologic was considered to be desirable and was subsequently included as an optional variable. The voting group also agreed that it was essential to include the reasons for discontinuation. These included lack of efficacy, serious adverse events (SAE), non-serious AEs, patient choice (side effects or other reasons), no reimbursement, patient non-compliance, or pregnancy.

#### 3.2.4 Current biologic therapy

The voting panel reached consensus that the name of the current biologic treatment, initiation date, dose, and dosing interval constitute essential variables for inclusion in the INVENT registry (Figure 5). It was also agreed that it was essential to include a list of pre-specified AEs associated with the current biologic. The voting group identified the following AEs as essential for inclusion: anaphylaxis, back or joint pain, conjunctivitis, eosinophilic granulomatosis with polyangiitis (EGPA), fever, hyper-eosinophilia (with and without symptoms), any SAE, skin symptoms, and swelling at the injection. In contrast, influenza-like symptoms, headache and muscle ache did not reach consensus.

#### 3.2.5 Biomarkers

Blood eosinophil count assessed without concomitant treatment with SCS or biologics and the date that it was determined were considered to be essential variables for inclusion by the voting group (Figure 6). In addition, specific IgE to aeroallergens or skin prick result tests were considered to be desirable and were included as optional variables. The voting group failed to reach a consensus on other biomarkers, although the expert panel recommended the inclusion of fractional exhaled nitric oxide (FeNO) and tissue eosinophilia from nasal polyp symptoms as optional variables.

#### 3.2.6 Comorbidities

Asthma, N-ERD and allergic rhinitis were also considered to be essential variables by the voting group and were considered mandatory for inclusion by the expert panel (Figure 7). Atopic dermatitis and eosinophilic otitis media were also considered to be essential by the voting group, although it is unclear if these data exist in all clinical settings. The voting group considered EGPA as an essential variable for inclusion, but this was excluded as a mandatory or optional variable by the expert panel, as EGPA does not fall within the scope of biologic prescribing by ENT specialists. The voting group failed to achieve consensus on other comorbidities and the expert panel agreed to exclude them from the registry.

#### 3.2.7 Asthma

The voting group agreed that use of inhaled corticosteroids (ICS) as a treatment for asthma and the number of courses of SCS in the past 12 months are essential variables and these were included by the expert panel as mandatory variables (Figure 8). The number of exacerbations or hospitalizations due to asthma and/or CRSwNP during the previous 12 months was considered to be desirable by the voting group and was included as an optional variable. In terms of asthma control, the voting group agreed that asthma control as measured by the Asthma Control Test (ACT; score 0–25) should be included, but they failed to reach consensus on asthma control as measured by the Asthma Control Questionnaire (ACQ)-5 or -7 (score 0–7). After discussion, the expert panel agreed to include level of asthma control via ACT and/or ACQ-5 as an optional variable. The voting group also failed to reach consensus on other asthma-related variables and the expert panel agreed to exclude these from the registry.

#### 3.2.8 CRSwNP-specific variables

The impact of nasal symptoms [measured by the Sino-Nasal Outcome Test 22 (SNOT-22), range 0–110], polyp size and extent [measured by the Nasal Polyp Score (NPS), range 0–8], nasal obstruction or congestion [measured by a visual analog scale (VAS), 0–10 cm], global sinonasal symptom burden (VAS, 0–10 cm) and loss of smell (VAS, 0–10 cm) were considered to be essential by the voting group and were included by the expert panel as mandatory variables for the INVENT registry (Figure 9). Subscores of the SNOT-22 questionnaire (rhinologic, ear/facial, sleep, and psychological) and rhinorrhea/post-nasal drip (VAS, 0–10 cm) were also considered to be essential or desirable variables and were included as optional variables by the expert panel. The voting group considered a semi-objective assessment of olfactory performance using Sniffin' sticks 12 (SST-12) to be desirable, but they failed to reach consensus on other measures of olfactory performance. Subsequently, the expert panel agreed to include SST-12, SST-16, University of Pennsylvania Smell Identification Test (UPSIT), and Brief Smell Identification Test (B-SIT) as optional variables. Facial pain/pressure and patient-reported outcomes (PROs) for CRSwNP disease control, including the Nasal Congestion Score, failed to reach consensus and were subsequently excluded from the registry.

#### 3.2.9 Follow-up variables

The voting group agreed that it is essential to include data from SNOT-22, NPS, a VAS for global sinonasal symptom burden or disease severity, a VAS for loss of smell and a measure of asthma control and these were subsequently included as mandatory variables (Figure 10). Although voting indicated that a PRO measure for CRSwNP was desirable, persistent uncertainty regarding its interpretation led the expert panel to exclude it from the registry.

#### 3.2.10 Validation survey

Overall, 18 individuals from 11 countries responded to the validation survey (Supplementary Figure S1). Results of the survey are shown in Supplementary Figures S2–S9 and indicate that most of the selected variables are available or can be obtained from the local registries. Some specific exceptions included occurrence of eosinophilic otitis media and the number of exacerbations or hospitalizations for asthma and/or CRSwNP, which appeared to be more difficult for the centers to obtain.

There is some inconsistency in measures used to assess asthma control, however the validation survey indicates that all centers have access to either ACT or ACQ. The level of severity of asthma will, when analyzed, be divided into subgroups. Similarly, the assessment of olfactory performance also differed, with a third of survey respondents unable to obtain data from SST-12 (0–12) or SST-16 (0–16), UPSIT (0–40) or B-SIT (0–12).

## 4 Discussion

This Delphi study successfully identified a consensus-based set of mandatory and optional variables for inclusion in INVENT, a registry study that aims to consolidate real-world data from across the world on the use of biologics in patients with CRSwNP. The final selected variables reflect expert agreement on the most clinically-relevant and feasible data to collect in routine clinical practice and are informed by both the literature and practical experience. Although there was no pre-determined focus on variables related to treatable traits, the Delphi identified variables that can change with treatment e.g., SNOT-22, NPS, VAS of upper airways diseases, and asthma scores. Importantly, both sense of smell and key biomarkers were also captured, with the loss of sense of smell assessed via both VAS and by objective measurements of olfactory performance, where available.

By engaging a diverse group of experts from across Europe and Australia, the Delphi process provided a structured approach to achieve international consensus and to ensure that the resulting dataset is both comprehensive and pragmatic. A validation survey was also conducted across all centers contributing to the INVENT registry to establish whether the variables that were agreed for inclusion can be obtained from the relevant national and regional registries. The results of this survey were consistent with the Delphi findings, indicating that the selected variables are generally available and align with those prioritized in the Delphi process. This supports the robustness of the methodology used and strengthens the foundation for a future registry capable of generating clinically meaningful insights.

The variables selected as mandatory align with known predictors of disease burden, biologic use and treatment response in CRSwNP. These include key demographic information, prior and current treatment history, relevant comorbidities and validated disease-specific measures such as SNOT-22, NPS and VAS assessments of nasal obstruction, global sinonasal symptom burden and loss of smell (25–27). Importantly, they also correspond with the five consensus-defined criteria for assessing response to biologic treatment in CRSwNP: reduced nasal polyp size, reduced need for SCS/salvage therapy, improved QoL (SNOT-22), improved sense of smell, and reduced impact of comorbidities (2, 23). It is generally agreed that both patient and physician reported outcomes should be included when evaluating overall disease burden and control (23). In addition, minimal clinically important difference thresholds have been established for a number of the registry variables, including SNOT-22, nasal congestion, loss of sense of smell, STT12/16 or UPSIT, NPS, and asthma-specific PROs (25, 28, 29). The inclusion of these variables in the INVENT registry will facilitate comparisons of real-world biologic effectiveness and enable monitoring of biologic-induced remission over time. The decision to include some variables as optional reflects a balanced approach that acknowledges both the potential clinical relevance of these data and the practical limitations of real-world data collection.

In this study, we found that most centers use similar variables for both screening and follow-up measurements, despite the lack of collaboration in developing their local registries. This is both impressive and reassuring as the majority of countries participating in the registry will be able to record most of the variables. This is a strength for the Delphi process, as we included more than nine countries, and also a strength for the future collaboration. This consistency supports the value of these findings and highlights their importance for the future development of international registries and associated analyses. These findings are relevant for both patients and healthcare authorities, as they can help guide future decision-making in this area. In particular, the data collected through the current process could support decisions on which new drugs to select, based on the most relevant variables.

Interestingly, some variables commonly reported in CRSwNP research, such as body mass index (BMI) (30), family history (31), and certain asthma-related metrics (32), failed to achieve consensus. Despite being frequently documented in patient histories or hospital records, and despite evidence from asthma studies showing an impact of obesity on response to biologics (33, 34), our Delphi survey results suggest that the experts considered BMI and family history to be less directly relevant for comparing biologic effectiveness. Instead, they preferred prioritizing variables with clearer implications for treatment evaluation and treatment decision-making.

This study has several limitations. Although the Delphi method promotes consensus, it is limited to the views of the selected experts and may not capture all perspectives, particularly from centers with limited access to biologics or different healthcare infrastructures. Furthermore, the focus on balancing variables that can be easily collected vs. those that are scientifically most relevant may have biased selection. Real-world data collection is inherently subject to the constraints of clinical practice and the variability among physicians involved in patient follow-up. The small number of respondents (*n* = 18) to the validation survey is an important limitation of our study. This limited sample may not fully capture the diversity of experiences across the broader target population. As a result, the conclusions from the feasibility assessment should be interpreted with caution, as they may not be generalizable to different settings, demographics or clinical contexts.

We also acknowledge that the absence of experts from the USA and the inclusion of only one expert from Australia in our Delphi panel may have influenced the final variable set. Patterns of clinical practice, including the choice of outcome measures and diagnostic tools, may vary across regions. This could partially explain the relatively low selection rate of UPSIT for olfactory assessment, as this tool is more commonly used in North America than in Europe.

In conclusion, the results of this Delphi study provide a robust foundation for the INVENT registry. By collating data from multiple regions, the INVENT registry has the potential to optimize use of biologics, support evidence-based decision-making, and ultimately improve outcomes for people living with CRSwNP.

## Data Availability

The raw data supporting the conclusions of this article will be made available by the authors, without undue reservation.
