# Supplementary figures and images for: Real-life evaluation of the effectiveness of biologics for chronic rhinosinusitis with nasal polyps in Europe: a Delphi study to define key variables for the INVENT registry
Source: Front Allergy. 2025 Oct 13;6:1680703. doi: 10.3389/falgy.2025.1680703 (PMC12554763; doi:10.3389/falgy.2025.1680703)

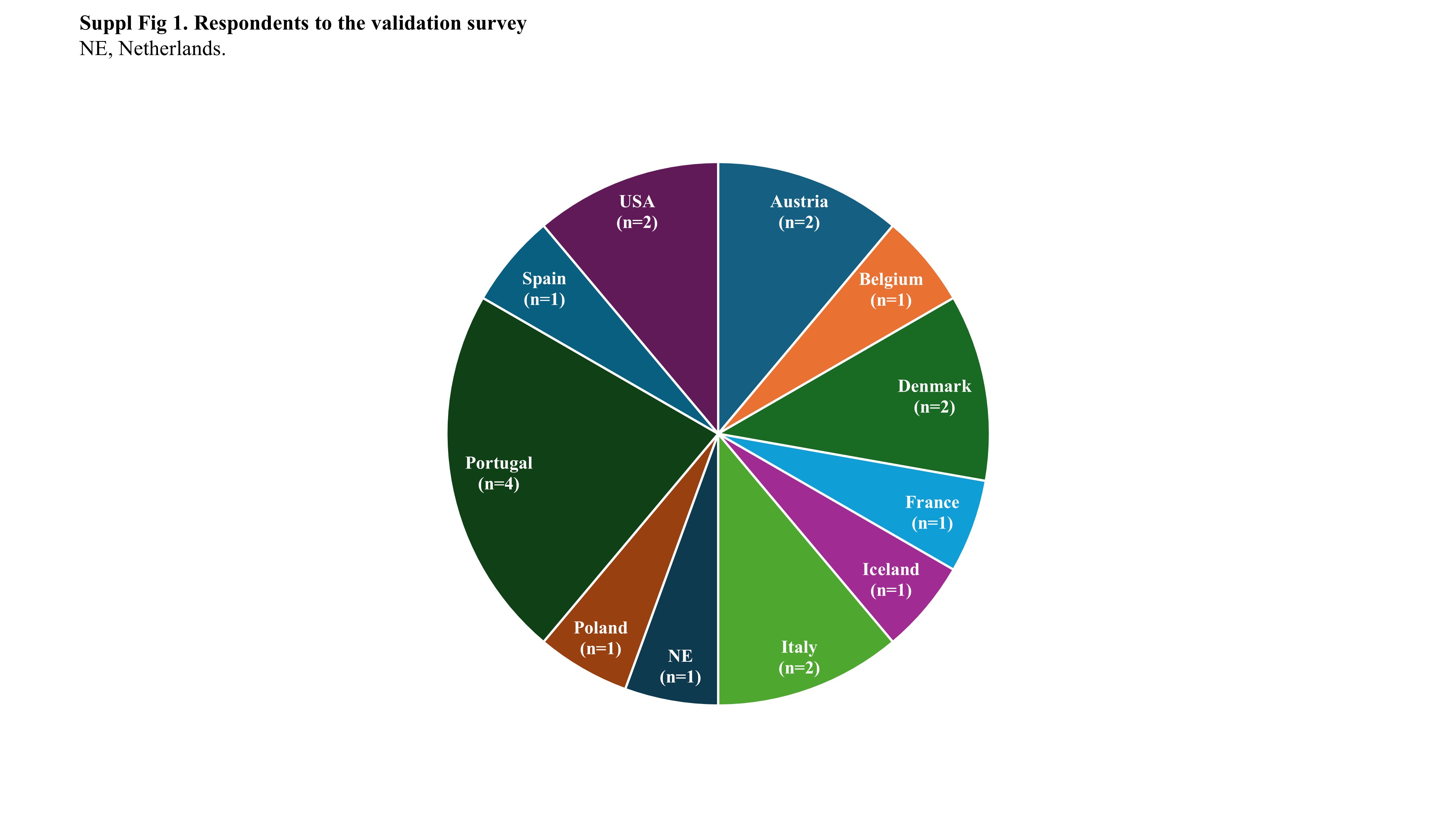

Supplement: Supplementary file 1 [file Image1.jpg]

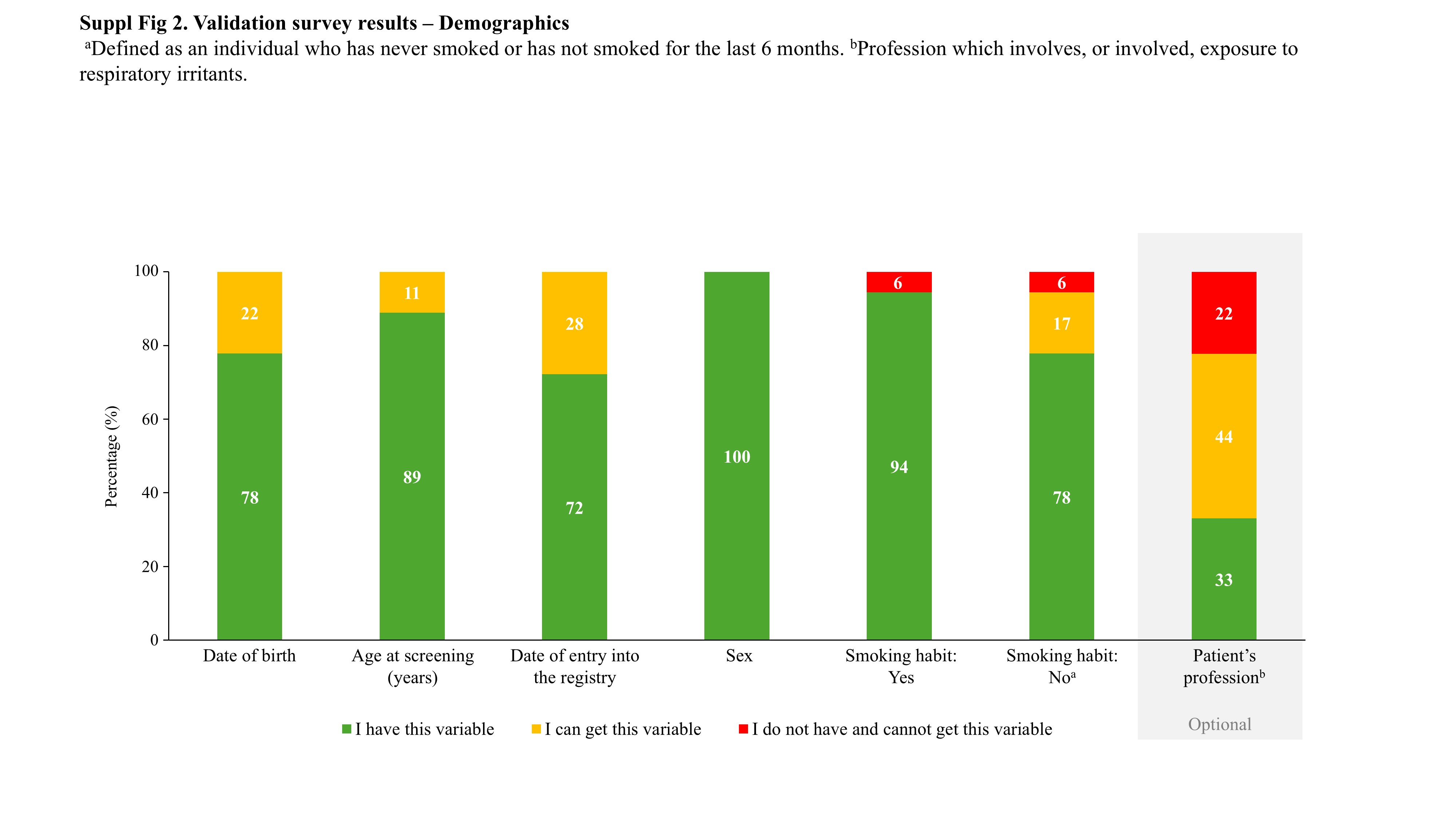

Supplement: Supplementary file 2 [file Image2.jpg]

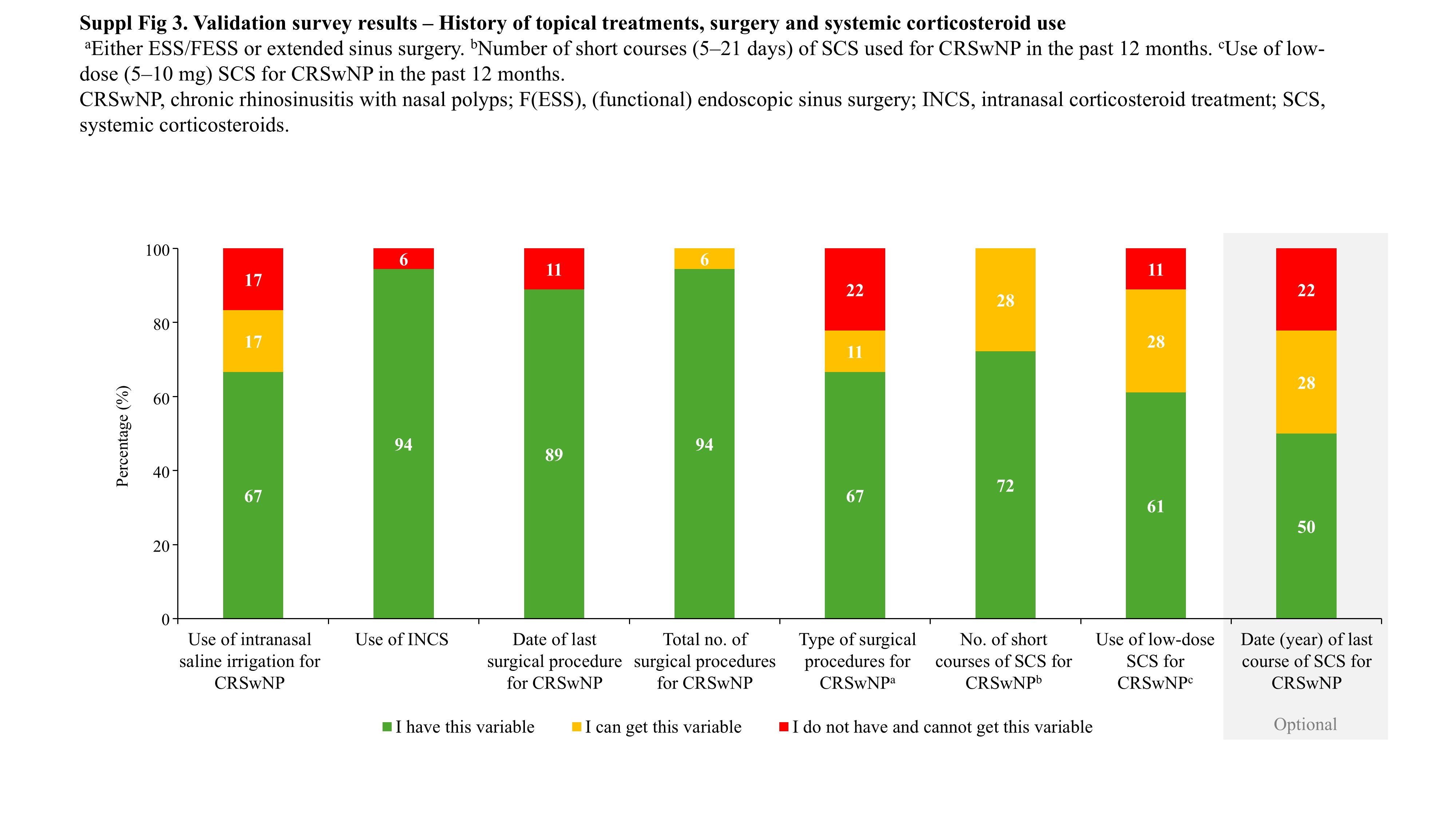

Supplement: Supplementary file 3 [file Image3.jpg]

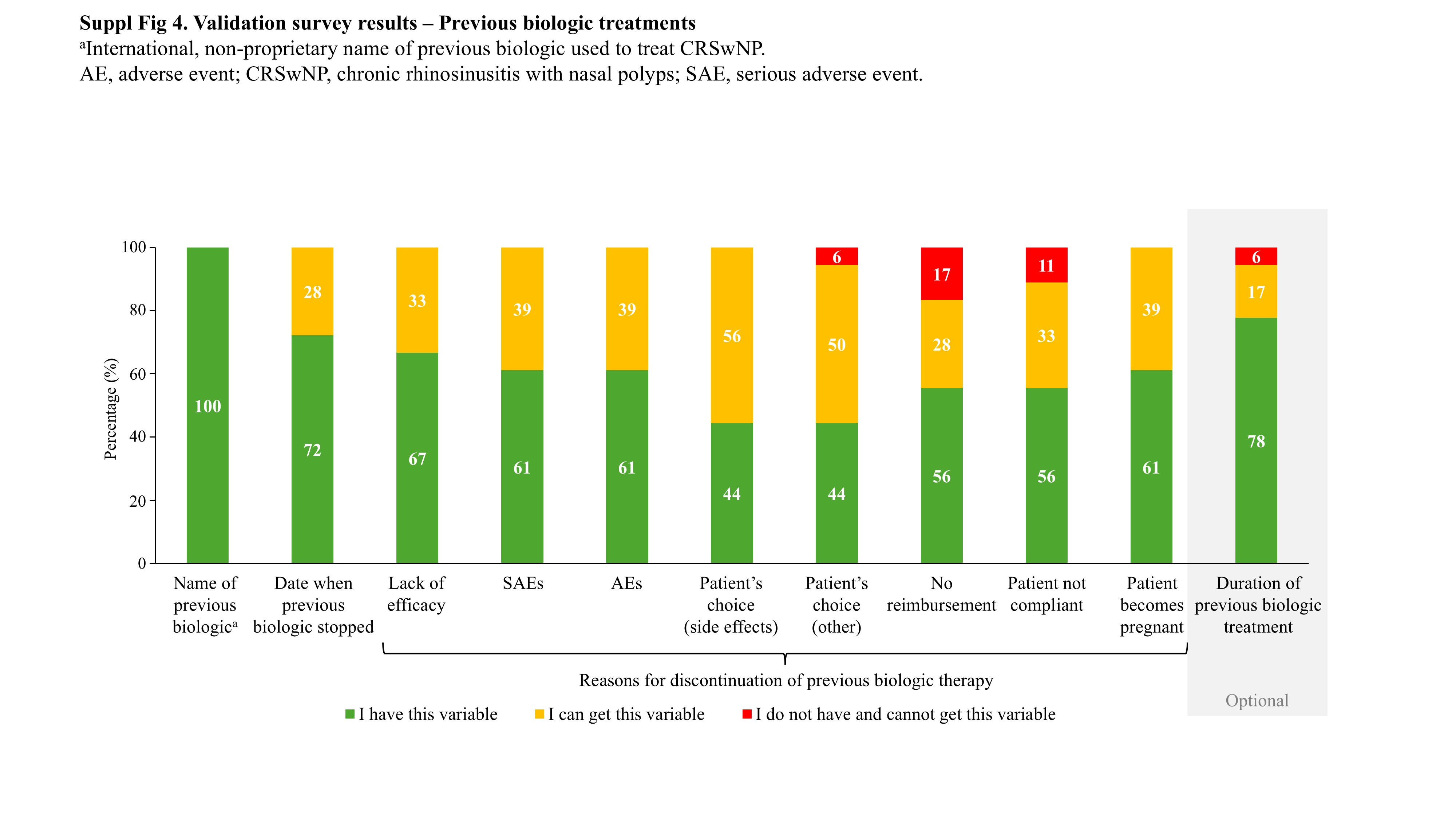

Supplement: Supplementary file 4 [file Image4.jpg]

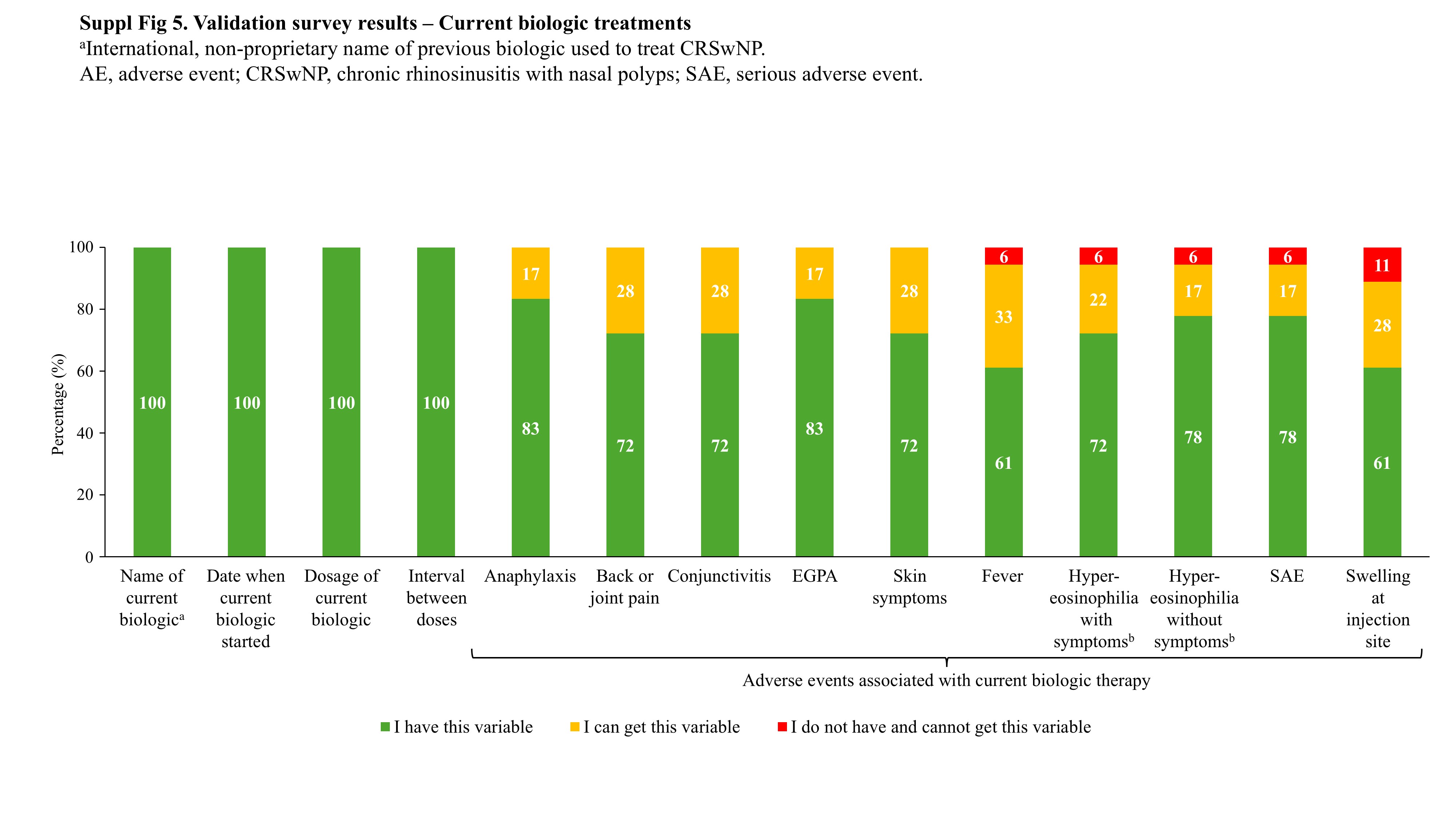

Supplement: Supplementary file 5 [file Image5.jpg]

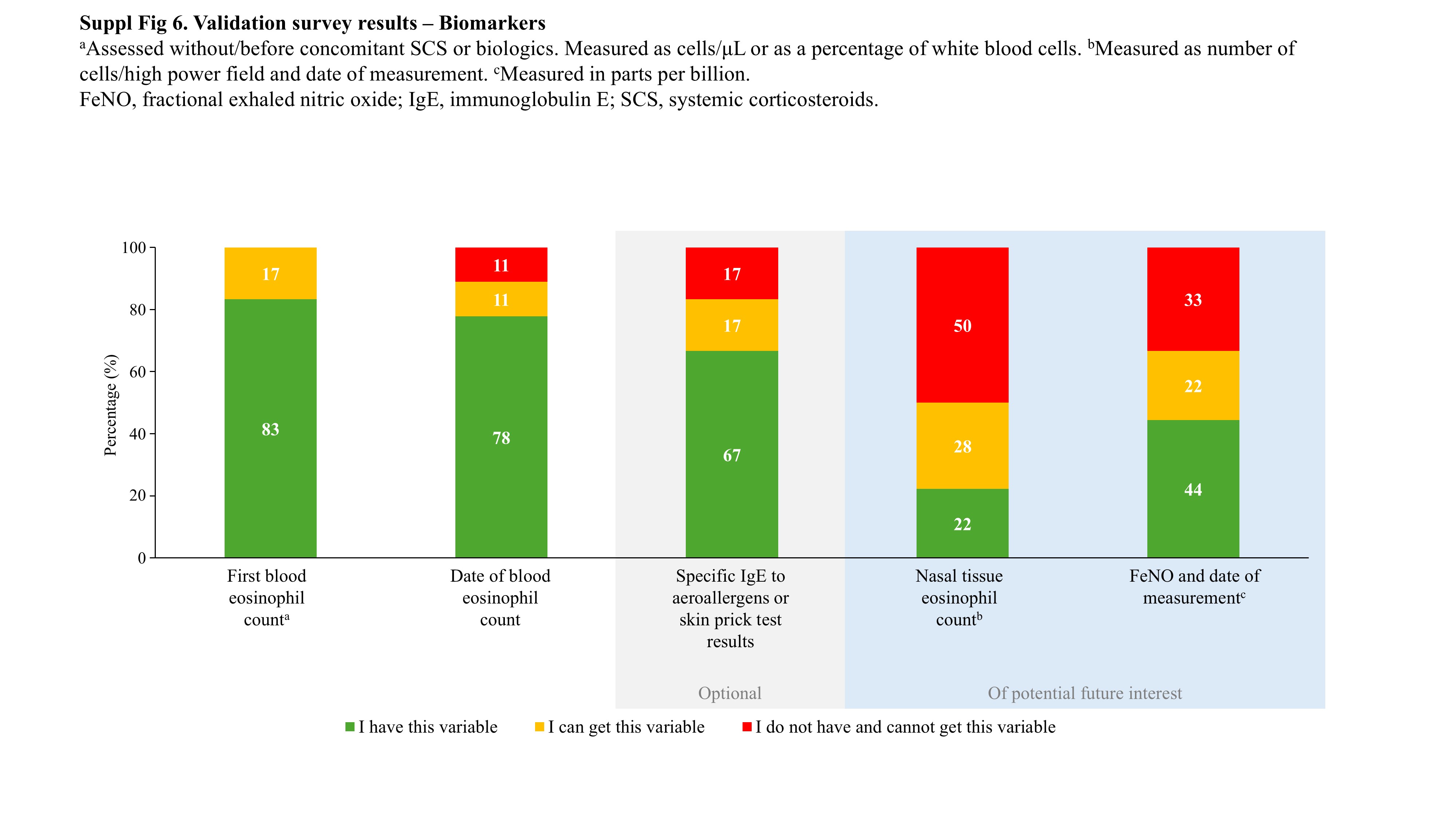

Supplement: Supplementary file 6 [file Image6.jpg]

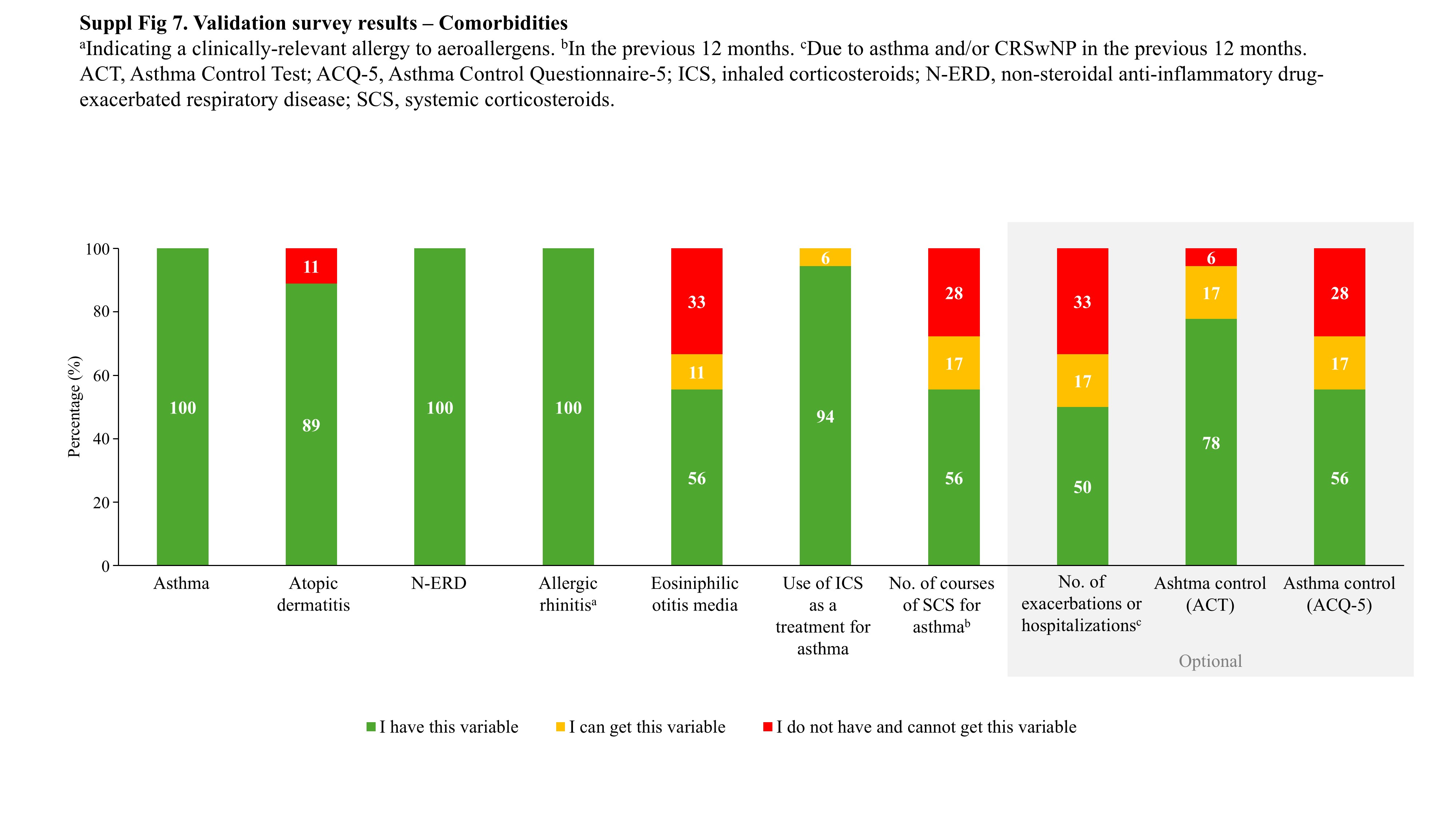

Supplement: Supplementary file 7 [file Image7.jpg]

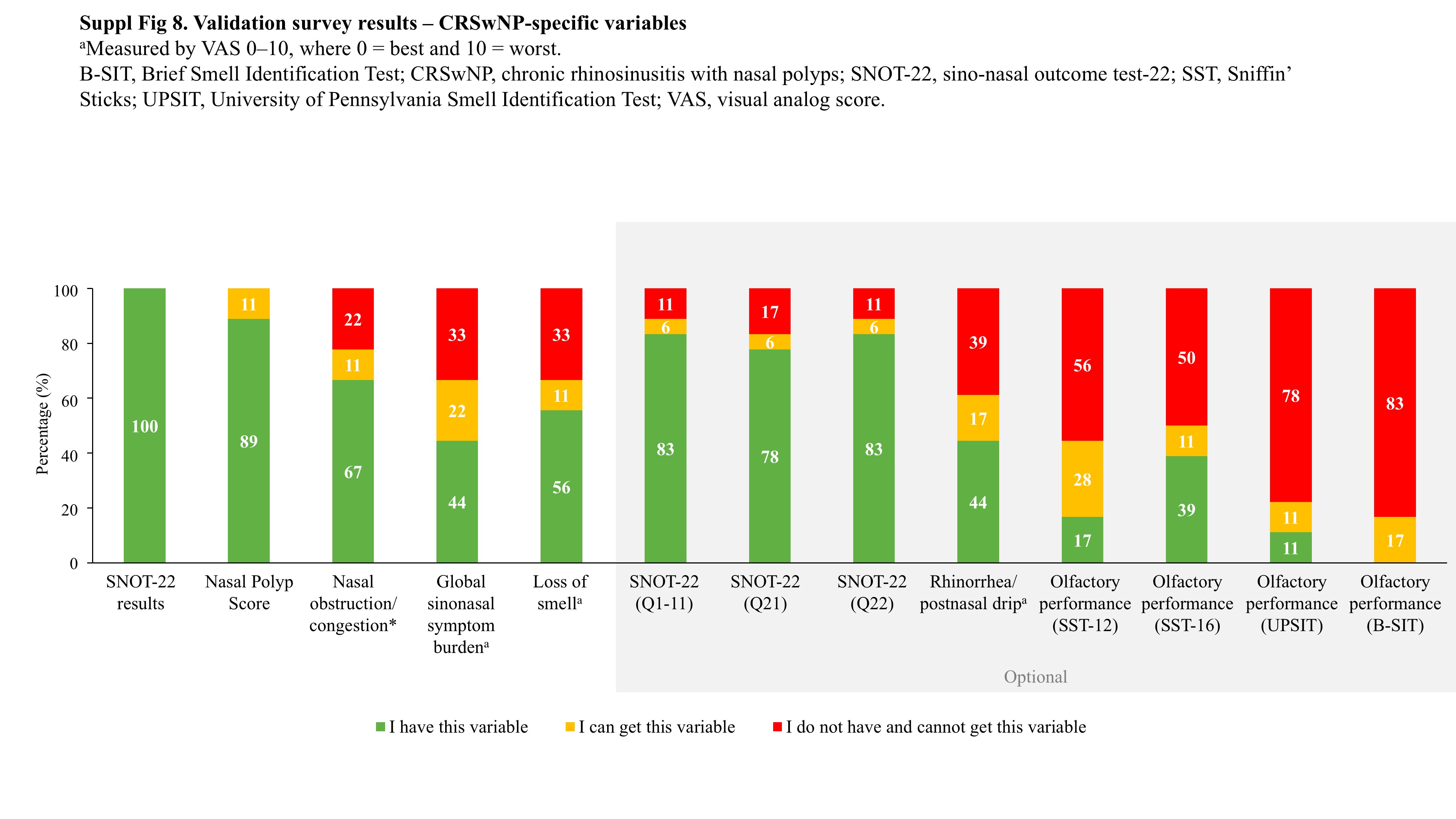

Supplement: Supplementary file 8 [file Image8.jpg]

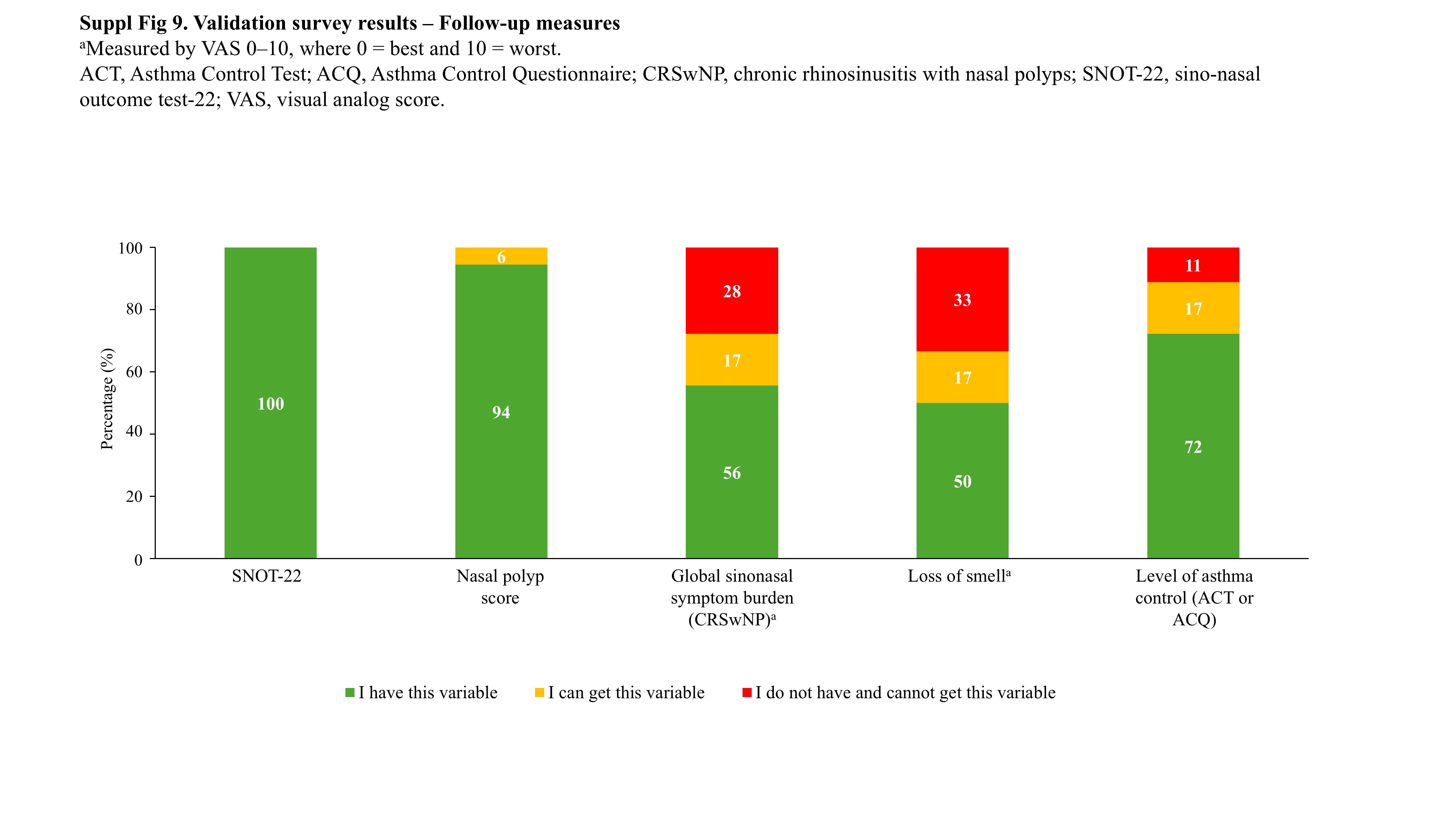

Supplement: Supplementary file 9 [file Image9.jpg]
